# Leguminous Seeds Powder Diet Reduces the Survival and Development of the *Khapra beetle*, *Trogoderma granarium* Everts (Coleoptera: Dermestidae)

**DOI:** 10.3390/biology9080204

**Published:** 2020-08-03

**Authors:** Spiridon Mantzoukas, Georgia Korbou, Alexandra Magita, Panagiotis A. Eliopoulos, Konstantinos Poulas

**Affiliations:** 1 Department of Pharmacy, School of Health Sciences, University of Patras, 26504 Patras, Greece; georgiakorbou@gmail.com (G.K.); alexandramag@hotmail.gr (A.M.); 2 Department of Agrotechnology, University of Thessaly, 45100 Larissa, Greece; eliopoulos@uth.gr

**Keywords:** diet, leguminous seeds, *Trogoderma granarium*, survival, development

## Abstract

Chemical storage pest control is interlinked with many challenges such as environmental pollution and toxicity to humans and animals. Alternative tools are thus being increasingly researched and applied to supplement and/or substitute old-fashioned chemical means. Entomotoxic proteins, such as the lectins of leguminous seeds, have been shown to be effective alternative control agents against many serious insect pests. The objective of this work was to evaluate the effect of the flour of three leguminous seeds, *Phaseolus vulgaris* L. (Fabaceae) (the common bean), *Vicia faba* L. (Fabaceae) (the broad bean) and *Glycine max* L. (Fabaceae) (the soya bean), against 4th instar larvae of *Trogoderma granarium* Everts (Coleoptera: Curculionidae). The flours were tested at different concentrations. They all demonstrated significant effects on larval mortality, as well as they all induced a decrease in the number of larvae reaching the pupal stage. The flours of *P. vulgaris* and *V. faba* were highly insecticidal against *T. granarium* larvae, especially at the highest concentrations (86.7% for PV100 and 90% for VF100). Our results enrich previous findings on the entomotoxic effect of leguminous plant lectins and highlight *P. vulgaris* and *V. faba* lectins as potential alternative control agents against *T. granarium*.

## 1. Introduction

Insects constitute the major pests of stored products whereby insect-related damage impacts the quality, quantity, and commercial and agronomic value of these food products. Many stored product pests are Coleopterans, and one of the most destructive and hard-to-kill species belong to the genus Trogoderma [1]. *Trogoderma granarium* Everts (Coleoptera: Dermestidae), also known as the khapra beetle, has been attributed the status of a quarantine organism [2] and has been classified among the most invasive species on a global scale [3]. It can survive extreme conditions for long periods of time, and it exhibits increasing resistance to several mainstream chemical insecticides as well as other non-chemical methods [4].

The challenges involved in the control of the khapra beetle are reinforced by the fact that chemical applications for pest control are problematic due to environmental pollution, emergence of pest resistance, toxicity to humans and related concerns, which account for the strict requirements imposed on their application on or near food, for safety reasons [5]. Chemical applications certainly remain one means of preventing some losses during storage. However, there is a need to explore new effective pest control methods which can confer adequate crop protection against insect pests, by employing a consumer- and environmentally friendly approach.

Plant lectins, which are carbohydrate-binding proteins, are such non-chemical control agents with proven insecticidal action against many insect taxa (for an extensive overview of the role and classification of plant lectins [6]). They are present mainly in seeds as well as in other tissues (bark, bulbs etc.), and they act as defense proteins against phytophagous insects [6,7]. Literature contains several studies documenting the effects of entomotoxic lectins of diverse plant species on the survival of a broad range of insect orders [8,9,10,11,12,13,14,15,16,17,18,19,20,21,22,23] with noteworthy results, inter alia, against Homoptera [8,24,25], Lepidoptera [9,26], and Coleoptera [10,27,28]. Indicatively, Macedo et al. [12,13]. reported 50% mortality of *Callosobruchus maculatus* F. (Coleoptera: Chrysomelidae) by lectins from *Koelreuteria paniculate* Laxm. (Sapindales: Sapindaceae) seeds, in the order of 0.7 and 0.3% (*w:w*), when added in artificial diets. The same entomotoxic lectins caused LD50 against *Ephestia kuehniella* (Lepidoptera: Pyralidade), albeit at 0.65% (*w:w*) [12]. Zero *Leptinotarsa decemlineata* (Coleoptera: Chrysomelidae) larvae reached the pupal instar stage when they were fed with the Gleheda solution, indicating that the entomotoxic Lamiacae lectin, which is structurally similar to the classical legume lectins, caused complete mortality [29]. Melander et al. [30] investigated the effect of entomotoxic lectins on the growth and survival of *Meligethes aeneus* larvae F. (Coleoptera: Nitidulidae) when fed *Brassica napus* L. (Brassicacae) anthers which had been soaked in 1% solution containing the lectins, with excellent results. Plant lectins can also be detrimental to insects with chewing and sap-sucking mouthparts such as aphids [31,32].

The objective of this study was to test the lectin insecticidal properties of three leguminous flours derived from seeds of *Phaseolus vulgaris* L. (Fabaceae: Faboideae), *Vicia faba* L. (Fabaceae: Faboideae) and *Glycine max* L. (Fabaceae: Faboideae) against 4th instar larvae of *T. granarium*. Legume lectins have provided encouraging output in terms of their insecticidal potential [33]. They make up a large family of homologous carbohydrate-binding proteins of non-immune origin which have been purified mostly from mature legume seeds although they have been also identified in other plant tissues [34,35,36,37]. To the best of our knowledge, however, this is the first time that these legumes, especially *V. faba*, or any plant lectins, have been tested against the khapra beetle. Our results are discussed in terms of enhancing the use of these alternative chemicals in stored-product Integrated Pest Management (IPM).

## 2. Materials and Methods

The initial stock of *T. granarium* was obtained from infested wheat. Insects were established in lab culture in a controlled environment chamber at 27 ± 3 °C and 73 ± 5% r.h., with alternating light ± cycles of 12 h. Larvae were kept in glass jars (0.25 l capacity) covered with muslin cloth. For their diet, they were provided with pesticide-free sterilized corn flour (200 g). Every two weeks emerged adults were carefully removed by sieving to use in experiments. Experimental insects were kept in a growth chamber (PHC Europe/Sanyo/Panasonic Biomedical MLR-352-PE), in controlled environmental conditions (27 ± 3 °C, 73 ± 5% r.h., light ± cycles of 12 h). These experimental insect larvae L_4_ were identified with their morphological traits of robust and hairy body, body length and dark brown color.

### 2.1. Flour from Leguminous Crop Seeds

The seeds of *P. vulgaris*, *V. faba* and *G. max* were reduced to flour in EMBIA Laboratory, Department of Pharmacy, University of Patras. For each of the three plants, the seeds (2000 g) were ground to obtain the flour, using the Waring Blender CB15TP (Waring Commercial, Huntington Beach, CA, USA). We then eliminated large particles by further sifting the flours with the Retch Jaw Crusher BB 50 sieve (mesh-size < 0.5 mm) (Retch GMBH, Haan, Germany). The different flour fractions were weighted using the Sartorius 126,400 scales (Sartorius AG, Gottingen, Germany), with precision of 0.01 g. All the flours were used directly in the biological assays.

### 2.2. Insecticidal Efficiency of the Flours of the Three Leguminous Seeds

The tested formulations were prepared by first placing the flours of the insecticidal legume seeds in sterile Petri dishes, in quantities which incrementally ranged every 2.5 g (from 0 g, 2.5 g, 5.0 g, 7.5 g to 10 g). To the leguminous seed flours, we also added corn flour to further supplement them. Corn flour quantities also ranged every 2.5 g, but they were provided in a decremental fashion (from 10 g, 7.5 g, 5.0 g, 2.5 g to 0 g) so that each Petri dish had the same overall content (10 g). The content of each Petri dish was homogenized by stirring it 20 times with a spatula. The Petri dish which contained only the corn flour constituted the control. In total, 10 Petri dishes were assembled, and 10 insects were placed in each Petri dish with 10 g of flour. Each Petri dish underwent 10 replications. Each treatment involved ten 4th instar larvae of *T. granarium*. Prior to the experiment, the larvae had been starved for a 24 h period. Daily observations of the Petri dishes were carried out for 16 days, by emptying the contents onto sterilized white paper to identify dead individuals. Forty-five days after the applications, we checked all Petri dishes and recorded the number of *T. granarium* pupae. Finally, after 60 days, we documented adult emergence.

### 2.3. Statistical Analysis

Corrected percent mortality was calculated using Abbott’s formula [38] and, prior to analysis, these values were arcsine transformed. Data were then analyzed by means of two-way ANOVA using the general linear model of the SPSS (SPSS FInc., Armonk, NY, USA, version 25) [39]. In case of significant F values, means were compared using the Bonferroni test. The Kaplan-Meier method was also selected to determine the mean overall survival of *T. granarium* individuals in each applied concentration, per treatment (flour) and exposure time. Comparison of median survival time was obtained using the Breslow test (Generalized Wilcoxon) (SPSS v.25.0).

The survival probability (which is also called the survivor- function), S(t), is the probability that an individual survives from the original time (e.g., beginning of treatment) to a specified future time, t. The following equation calculates the proportion of initial individuals which are still alive at time t:

S(t) = e ^−^^τ/^^μ^

We also used the Cox regression model to calculate the *hazard function* [40]. The latter is denoted by h(t) and it allows us to calculate the risk of dying at time *t* while evaluating the effect of several simultaneous factors on survival. The hazard function is estimated as follows:

h(t) = h0(t) × exp (b1 × 1 + b2 × 2 + …+ bp × p),

where by t denotes the survival time; h(t) represents the hazard function which is determined by a set of covariates; (b1, b2, …, bpb1, b2, …, bp) symbolize the coefficients which measure the impact of covariates; h0 is called the baseline hazard.

## 3. Results

All the flours derived from the seeds of the three edible leguminous crops demonstrated noteworthy insecticidal action against the *T. granarium* larvae. However, the flours tested were not equally toxic to the beetles. Toxicity increased as the concentration in seed flour increased. The flours of *P. vulgaris* and *V. faba* were the most effective against *T. granarium*. More specifically, with the flour of *V. faba*, the median lethal time values varied from 8.87 (VF100) to 12.2 days (VF25) (Table 1), with the flour of *P. vulgaris*, the median lethal time values varied from 9.03 (PV100) to 12.7 days (PV25) (Table 1), and, finally, with the flour of *G. max*, the median lethal time values varied from 9.13 (GM100) to 13.1 days (GM25) (Table 1) (Chi-square = 24.999, df = 1, *p* < 0.001, Breslow (Generalized Wilcoxon)). The control median lethal time was 15.6 days.

**Figure 1:**
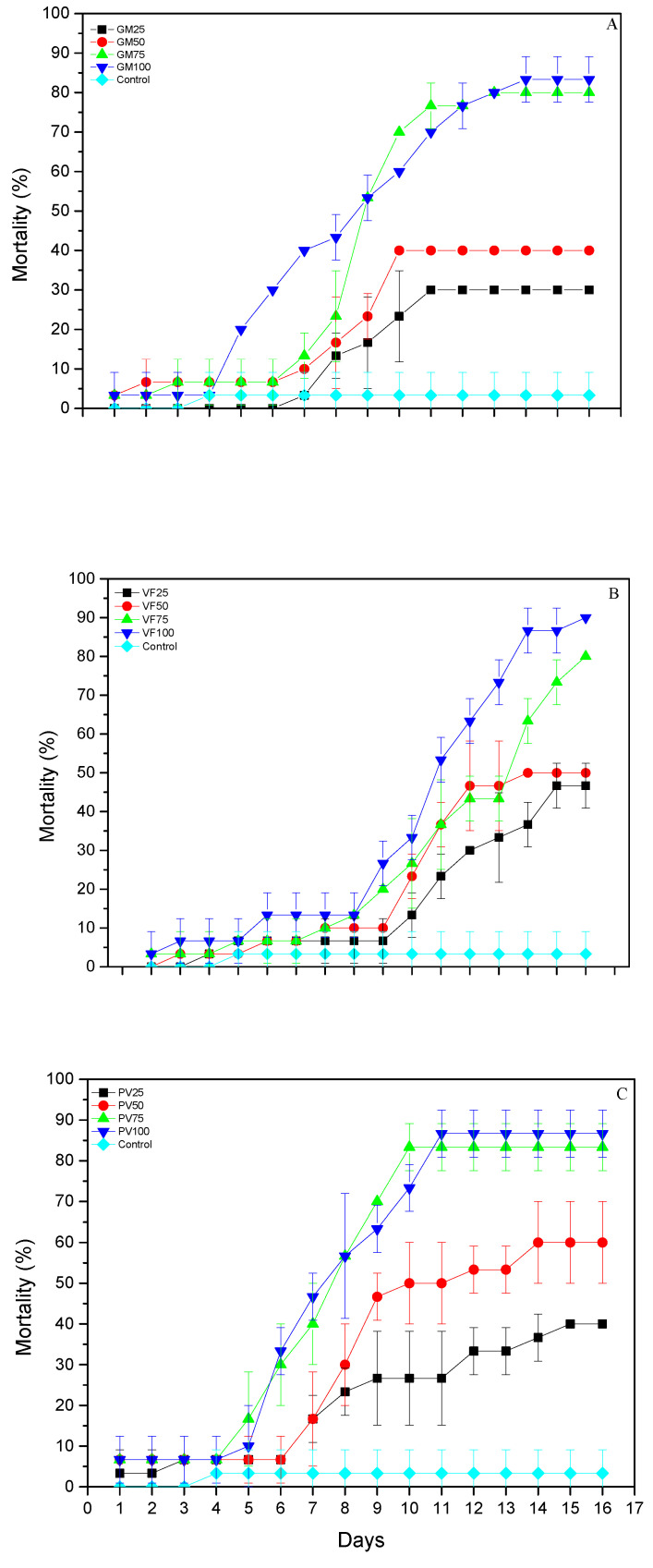
Mortality (% ± sd) of *T. granarium* larvae exposed to (**A**) *G. max* flour, (**B**) *V. faba* flour and (**C**) *P. vulgaris* flour after 16 days.

In terms of mortality, all main effects and associated interactions between the three leguminous flours were significant, for flour (F = 3.079, df = 12.623; *p* < 0.001), for exposure time (F = 12.666, df = 15.623; *p* = 0.0001), and for flour × exposure time (F = 1.773; df = 180.623; *p* = 0.0001). After 16 days, control mortality was 3.3%. On the *G. max* flour, it ranged between 30 (GM25) and 83.3% (GM100) (Figure 1A); on the *V. faba* flour, it ranged between 46.7 (VF25) and 90% (VF100) (Figure 1B); on the *P. vulgaris flour*, mean larval mortality ranged between 40 (PV25) and 86.7% (PV100) (Figure 1C).

Table 1 presents the number of *T. granarium* pupae 45 days post treatment. The number of pupae was also dependent upon the applied concentrations. Indeed, there was a significant difference between the number of treated pupae (F = 1.688, df = 12.632, *P* < 0.001) and the number of control pupae. After 60 days, there was also a significant difference between the number of treated and untreated adults (F = 1.989, df = 12.632, *p* < 0.001).

The treatments that reinforced the mortality of *T. granarium* larvae had positive β-values (Table 2). However, their influence is estimated in connection with the total number of dead larvae. The treatments which had a progressively greater mortality effect were PV 75, PV 100, VF 75, VF 100, GM 75, and GM 100 (Table 1). The Hazard Rate (Exp(B)) in these treatments was higher than all treatments and the control. Finally, the above treatments were statistically significant, with *p* values of 0.006, 0.002 and <0.001 respectively (Table 2).

## 4. Discussion

The objective of this work was to evaluate the effect of the flour of three leguminous seeds, *P. vulgaris*, *V. faba* and *G. max* against 4th instar larvae of *T. granarium*. All flours demonstrated a significant dose-dependent effect on larval mortality as well as on pupation rate. However, mortality induced by the flours of *P. vulgaris* and *V. faba* exceeded that of *G. max*, especially at the highest concentrations (86.7% for PV100 and 90% for VF100).

Entomotoxic lectins have been reported plentiful in the seeds of legumes, including bean and soybean. [11] They have been extensively studied for their significant entomotoxic properties pertaining to a broad spectrum of insect orders which attack a wide range of important crops (including wheat, rice, tobacco, and others) [41,42]. For instance, lectins from the seeds of *Canavalia brasiliensis* (Fabaceae: Faboideae), *Dioclea grandiflora* (Fabaceae: Faboideae), *Dioclea rostrate* (Fabaceae: Faboideae), *Cratylia loribunda* (Fabaceae: Faboideae), and *P. vulgaris* plants contain lectins which have successfully been assayed for their protection of seeds against the beetle *Callosobruchus maculatus* F. (Coleoptera: Chrysomelidae) [41].

Lectins can interfere with important physiological insect functions either by binding to sugars on the surface of glycoprotein-rich epithelial gut cells, thus affecting nutrient absorption, or by becoming internalized and finding targets intracellularly, thereby disturbing metabolic pathways. A significant prerequisite is that lectins first bypass being degraded by the digestive enzymes of the insect gut. Most insecticidal lectins appear resistant to proteolysis as well as tolerant in a broad pH spectrum. The cellular pathways affected will vary in relation to insect species and lectin type [41,42,43].

The array of plant lectin-induced effects involves significant mortality, delays in insect development, reduced pupation, fecundity and/or adult emergence, susceptibility to natural enemies and other [11,12,13,19,20,21,22,23,44]. Some of the aforementioned complications are confirmed in our study documenting significant mortality as well as a reduction in the final number of individuals completing their biological cycle after being fed with the bean flour and the fava bean flour; this could be explained by the lectin action which limits food consumption and, consequently, halts insect development. The reduction in the production of progeny in the treated substrate is deemed equally important as parental mortality, if not more so. In a similar vein, *Hypera postica* Gyllenhal (Coleoptera: Curcuniolidae) larvae died swiftly after feeding on bean flour [28]. The black bean flour introduced into the millet’s flour inhibited the development of the red weevil of flour and *Sitophilus oryzae* L. (Coleoptera: Curculionidae) [45,46]. SBA (soybean agglubitin) had a negative impact on larvae of the melon fly *Bactrocera cucurbitae* (Coquillet) (Diptera: Tephritidae) in terms of pupal weight, number of pupae and number of emerging insects, as lectin concentration increased [47]. *Callosobruchus maculatus* which is detrimental to the chickpea *Cicer arietinum* (Fabaceae: Faboideae) could not develop in the seeds of *Phaseolus* spp. In fact, the bean meals disrupted female fecundity, adult emergence, and the developmental timespan [48].

The different degrees of insecticidal efficiency between the three leguminous flours of the present study must be linked to their botanical group. Observed differences in their entomotoxicity can be attributed to their taxonomy as well as to the variability in the chemical and biochemical constituents [11,45,46,49]. Differences in lectin entomotoxicity could also be accounted for by the volume and spatial arrangement of their carbohydrate recognition domain (CRD) [50].

Edible legumes containing entomotoxic lectins should be considered in pest management strategies. They could also provide an alternative to introducing entomotoxic factors in the genome of plants which is otherwise prohibitive considering the cost of seeds. Based on our experimental results, the bean flour and the fava bean flour which caused the highest mortality to *T. granarium* larvae could be considered in the framework of storage infestation control.

## 5. Conclusions

Edible legumes containing entomotoxic lectins appear promising for use in pest management strategies. They could also provide an alternative to introducing entomotoxic factors in the genome of plants which is otherwise prohibitive considering the cost of seeds. Based on our experimental results, the bean flour and the fava bean flour which caused the highest mortality to *T. granarium* larvae could be considered in the framework of storage infestation control.

## Figures and Tables

**Table 1:** Mean effect of *P. vulgaris*, *V. faba* and *G. max* flour on pupation, adult emergence, and median survival time of larval instars of *T. granarium*. Means sharing the same lower-case letters are not significantly different from each other.

Treatment	Pupation (% ± sd)	Adult Emergence (% ± sd)	Median Lethal Time (days ± sd) (Chi-square = 24.999, df = 1, *p* < 0.001 Breslow (Generalized Wilcoxon))
**PV 25**	69.2 ± 0.9 b	32.8 ± 3.8 c	12.7 ± 0.3 b
**PV 50**	53.8 ± 1.8 d	18.7 ± 3.7 d	11.4 ± 0.2 c
**PV 75**	3.5 ± 1.7 g	0 ± 0 e	10.4 ± 0.5 d
**PV 100**	2.3 ± 2.4 g	0 ± 0 e	9.03 ± 0.2 e
**VF 25**	61.6 ± 2.9 c	48.6 ± 2.7 b	12.2 ± 0.4 b
**VF 50**	32.7 ± 1.8	26.6 ± 3.7 c	10.8 ± 0.3 d
**VF 75**	2.9 ± 0.8 g	0 ± 0 e	9.25 ± 0.4 e
**VF 100**	0.8 ± 0.7 g	0 ± 0 e	8.87 ± 0.1 e
**GM 25**	63.2 ± 4.8 b	31.4 ± 2.7 c	13.1 ± 0.1 b
**GM 50**	39.7 ± 4.6 e	18.3 ± 1.3 d	10.3 ± 0.3 d
**GM 75**	3.8 ± 1.7 g	0 ± 0 e	9.89 ± 0.2 e
**GM 100**	0.8 ± 0.2 g	0 ± 0 e	9.13 ± 0.3 e
**Control**	92.7 ± 1.5 a	87.7 ± 4.9 a	15.6 ± 0.2 α

**Table 2:** Toxicity variables in the equation from Cox regression for the treatments against larval instars of *T. granarium.* All tested treatments had 1 *df.*

Treatment	B ^†^	Std	Sig	Exp(B) ^††^	95.0% CI for Exp(B)	
					Lower	Upper
**PV 25**	−1.377	0.375	0.530	0.252	0.121	0.526
**PV 50**	−1.028	0.375	0.379	0.358	0.172	0.745
**PV 75**	0.176	0.280	0.006	1.192	0.688	2.065
**PV 100**	0.252	0.287	0.000	1.287	0.733	2.258
**VF 25**	−0.635	0.310	0.040	0.457	0.289	0.973
**VF 50**	−0.783	0.327	0.017	0.530	0.241	0.868
**VF 75**	0.084	0.327	0.002	0.909	0.645	1.575
**VF 100**	0.096	0.290	0.002	0.957	0.741	1.867
**GM 25**	−1.480	0.390	0.892	0.228	0.106	0.488
**GM 50**	−1.088	0.352	0.792	0.337	0.169	0.672
**GM 75**	0.038	0.283	0.002	0.927	0.526	1.633
**GM 100**	0.076	0.289	0.002	0.962	0.556	1.676
**Control**	−13.971	157.535	0.929	0.000	0.000	1.063E+128

**^†^B: B** values are associated with increased hazard and decreased survival time; as the predictor increases, the hazard of the event increases and the predicted survival duration decreases. Negative coefficients indicate decreased hazard and increased survival times. **^††^Exp(B):** the ratio of hazard rates.
